# Exercise as the Key to Improve Cardiopulmonary Function in Patients with Valvular Heart Disease: A Systematic Review and Meta-Analysis

**DOI:** 10.31083/j.rcm2408237

**Published:** 2023-08-17

**Authors:** Liqing Zeng, Peng Pi, Peizhen Zhang, Yu Zhu, Lumeng Yang, Chen Wang

**Affiliations:** ^1^School of Sports Medicine and Rehabilitation, Beijing Sport University, 100084 Beijing, China; ^2^Department of Rehabilitation Medicine, Linfen Central Hospital, 041000 Linfen, Shanxi, China

**Keywords:** exercise, valvular heart disease, cardiopulmonary function, cardiac rehabilitation, quality of life

## Abstract

**Background:**

Valvular heart disease (VHD) is a type of cardiovascular disease with abnormal heart valve structure and/or function and a rapidly growing cause of global cardiovascular morbidity and mortality. Physical inactivity is a problem for patients with VHD, especially after surgery. However, there is no data on the effects of exercise on VHD from large multicentre randomised controlled trials (RCTs). Therefore, we conducted a systematic review and meta-analysis to provide a comprehensive analysis of small RCTs to evaluate the effects of exercise on cardiopulmonary function in patients with VHD and provide an evidence-based medicine basis for developing and guiding the clinical application of exercise in patients with VHD.

**Methods:**

We conducted a systematic review and meta-analysis of RCTs. We systematically searched electronic databases (PubMed, Web of Science, Embase, Cochrane Central Register of Controlled Trials, China National Knowledge Infrastructure [CNKI], China Science and Technology Journal Database [VIP], WanFang Database, and SinoMed [CBM]) for all studies on exercise and VHD from their inception to January 2023. The quality of included studies was assessed using the Cochrane risk-of-bias tool. The primary outcomes were the six-minute walk test distance (6MWD), left ventricular ejection fraction (LVEF), and short-form 36-item health survey (SF-36).

**Results:**

This systematic review included 22 RCTs with 1520 subjects (869 men and 651 women). The meta-analysis results showed that exercise significantly improved exercise capacity measured by the 6MWD (mean difference [MD] = 25.54, 95% confidence interval [CI] = 19.98–31.11, *$I^{2}$* = 0%, *p* < 0.00001), LVEF (MD = 6.20, 95% CI = 4.76–7.65, *$I^{2}$* = 66%, *p* < 0.00001), and quality of life measured by the SF-36 (physical function: MD = 3.42, 95% CI = 2.12–4.72, *$I^{2}$* = 12%, *p* < 0.00001; mental health: MD = 3.86, 95% CI = 0.52–7.20, *$I^{2}$* = 68%, *p* = 0.020; social function: MD = 2.30, 95% CI = 0.64–3.97, *$I^{2}$* = 45%, *p* = 0.007; bodily pain: MD = 2.60, 95% CI = 0.83–4.37, *$I^{2}$* = 22%, *p* = 0.004) in patients with VHD compared to healthy controls.

**Conclusions:**

This study suggests that exercise can significantly improve cardiopulmonary function, enhance physical and social function, reduce bodily pain, and potentially improve mental health in patients with VHD, providing an evidence-based basis for better recovery in patients with VHD.

## 1. Background

Valvular heart disease (VHD) is a type of cardiovascular disease (CVD) with abnormal heart valve structure and/or function and is a rapidly growing cause of global cardiovascular morbidity and mortality [1, 2, 3]. VHD’s epidemiology varies substantially worldwide, with a predominance of functional and degenerative diseases in high-income countries and rheumatic heart disease (RHD) in low- and middle-income countries [4, 5]. RHD remains the most common VHD manifestation worldwide, affecting approximately 41 million individuals [6]. In contrast, calcific aortic stenosis (AS) and degenerative mitral valve disease affect 9 and 24 million individuals, respectively [4]. The Global Burden of Disease Report states that approximately 0.49% of the global population has RHD, which is likely to be an underestimate due to limited global data, underdiagnosis, and the absence of a formal reporting system [7, 8].

Acquired diseases of the aortic and mitral valves are the most common cause of morbidity and mortality among VHDs [9, 10]. Aortic valve disease (AVD) is the most common cause of mortality and the reason for procedural intervention among the various VHD types. Mortality due to AVD has steadily increased from the 1970s to the 2000s, with an annual increase of approximately 1.6% [11]. In addition, AS prevalence is increasing in ageing populations and carries significant risks [12]. If left untreated, symptomatic severe AS can have a mortality of 75% at 3.5 years, with up to 50% of those affected dying suddenly [13]. Based on population growth estimates and the assumption of stable mortality, the number of deaths attributable to VHD is expected to double by 2030, likely driven by AVD [9]. Therefore, how to improve the status quo of patients with VHD is a significant social issue.

As the concept of ‘exercise is medicine’ gains influence worldwide, exercise is gaining a foothold in treating CVD [14, 15]. Physical inactivity is common in patients with VHD, especially after surgery. The patients may experience presurgical dyspnoea and physical incapacity, immobilisation during hospitalisation, and potential postsurgical complications and restrictions due to the healing of the sternum [16, 17]. Therefore, patients with VHD tend to have reduced cardiopulmonary function, lowering their exercise capacity and ultimately affecting their quality of life. A recent European position paper stated that cardiac rehabilitation (CR), including exercise training, should be available to all patients after heart valve surgery [18, 19]. Historical data suggest that exercise training is an effective and fundamental element of CR [20]. Many potential benefits of CR are due to exercise, including increased maximal oxygen uptake, improved endurance, improved endothelial function, reduced myocardial blood flow reserve, reduced body weight, and further improved lipid and blood pressure control [20].

The importance of exercise is evident. A primary outcome of any exercise programme is measuring individuals’ response to training, such as functional capacity or cardiorespiratory fitness (CRF) [21]. In addition, improving functional capacity cannot be separated from enhancing cardiopulmonary function [21]. The six-minute walking test (6MWT) is a widely available and well-tolerated test for assessing the functional capacity of patients with CVD [22]. Reduced functional capacity in patients with CVD has been associated with a worse prognosis and an increased socioeconomic burden. It has been the target of various medical and interventional treatment modalities [22]. The 6MWT is also a very useful, reliable, and valuable tool for indirectly assessing CRF, which reflects cardiopulmonary function [23]. Studies have shown that each metabolic equivalent increase in fitness may confer a 12% decrease in mortality [24].

Patients who undergo surgery are often severely deconditioned and are therefore likely to particularly benefit from exercise interventions that improve their cardiopulmonary function, physical integrity, mobility, and ease in performing daily living activities [25, 26]. Exercise has been shown to improve outcomes in patients with various cardiac disorders. However, no specific recommendation is available about how exercise training should be conducted for perioperative patients with VHD [27, 28]. Currently, exercise for patients with VHD generally follows guidelines based on expert consensus [29, 30, 31]. Therefore, we conducted a systematic review and meta-analysis to provide a comprehensive analysis of small randomised controlled trials (RCTs) [13, 18, 27, 32, 33, 34] to evaluate the effects of exercise on cardiopulmonary function in patients with VHD and provide an evidence-based medicine basis for developing and guiding the clinical application of exercise in patients with VHD.

## 2. Materials and Methods

This study was registered in the International Prospective Register of Systematic Reviews (PROSPERO) (CRD42023401698) and performed according to the Preferred Reporting Items for Systematic Reviews and Meta-Analysis (PRISMA).

### 2.1 Study Eligibility Criteria

#### 2.1.1 Inclusion Criteria

Studies that met the following criteria were included: (i) an RCT whose study population was patients diagnosed with VHD; (ii) the patients in the experimental group received exercise therapy, including the exercise intervention component of CR; (iii) the patients in the control group received only conventional treatment without an exercise program; (iv) the primary outcome indicators included 6MWT distance (6MWD), left ventricular ejection fraction (LVEF), the short-form 36-item health survey (SF-36), maximal or peak oxygen uptake (${\mathrm{VO}}_{\text{2max}}$/${\mathrm{VO}}_{\text{2peak}}$); (v) the data relating to the outcome indicators were complete and available.

#### 2.1.2 Exclusion Criteria

Studies that met the following criteria were excluded: (i) no randomised control group or a randomised control group that was not given conventional treatment; (ii) no exercise-related interventions in the experimental group; (iii) duplicated published trial data; (iv) was a non-clinical trial, review, or conference paper; (v) the full text was unavailable.

### 2.2 Search Methods for Identifying Studies

Articles were searched for in eight electronic databases (PubMed, Web of Science, Embase, Cochrane Central Register of Controlled Trials [CENTRAL], Chinese National Knowledge Infrastructure [CNKI], China Science and Technology Journal Database [VIP], WanFang Database, and SinoMed [CBM]) from inception to January 2023 using the following search term: ‘exercise OR aerobic exercise OR cardiac rehabilitation’ AND ‘valvular heart disease’ AND ‘randomised controlled trial OR randomised OR placebo’.

### 2.3 Study Selection and Data Extraction

The study selection process was divided into two stages. First, three reviewers (LQZ, PP and LMY) independently screened the studies based on their titles and abstracts. Second, eligible articles were selected by reading their full text. Any disagreements between the three reviewers in the above steps were resolved by consulting a superior expert (PZZ).

The reviewers (LQZ, PZZ and YZ) independently performed the data extraction. They primarily extracted the following information from the included studies: the first author’s name, publication year, location, duration and type of intervention, the participants’ characteristics (number, sex, and age), and the outcome indicators. The extracted data are summarised in Table 1 (Ref. [13, 18, 27, 32, 33, 34, 35, 36, 37, 38, 39, 40, 41, 42, 43, 44, 45, 46, 47, 48, 49, 50]).

**Table 1.** **Characteristics of included studies**.

Study	Location	Intervention duration	Type	Group	N	Sex (M/F)	Age (years)	Ending indicators	Type of exercise	Exercise frequency
Hegazy *et al*. (2021) [37]	UAE	8 weeks	MVRS	EG: ET	100	25/25	39 ± 4.28	LVEF, 6MWT	inspiratory muscle training, aerobic exercise	8 t/w
				CG: CT		26/24	38.3 ± 3.29			
Li *et al*. (2020) [50]	China	7 days	MVRS	EG: ET	80	22/18	56.34 ± 2.52	LVEF, 6MWT, SF-36	1–2 level: MOTO Med 3–4 level: aerobic exercise	7 t/w
				CG: CT		23/17	56.15 ± 2.62			
Zheng (2020) [47]	China	7 days	MVRS	EG: ET	54	15/12	49.3 ± 5.2	LVEF, 6MWT, SF-36	1–2 level: MOTO Med 3–4 level: aerobic exercise+ resistance training	7 t/w
				CG: CT		16/11	49.2 ± 5.1			
Sun *et al*. (2021) [43]	China	7 days	MVRS	EG: ET	200	54/46	69.1 ± 2.7	6MWT, SF-36	1–2 level: MOTO Med 3–4 level: aerobic exercise+ resistance training	7 t/w
				CG: CT		56/44	70.1 ± 3.8			
Sibilitz* et al*. (2016) [27]	Denmark	12 weeks	MVRS	EG: ET	147	59/13	62 ± 11.5	LVEF, 6MWT, SF-36	aerobic exercise, lower extremity strength training	3 t/w
				CG: CT		53/22	61 ± 9.9			
Lindman *et al*. (2021) [35]	USA	6 weeks	TAVR	EG: ET	50	17/8	76 ± 7	LVEF, 6MWT	walking, resistance training	6–30 t/w
				CG: CT		16/9	76 ± 9			
Scordo (1991) [36]	USA	12 weeks	MVP	EG: ET	38	0/19	34 ± 6	${\mathrm{VO}}_{\text{2max}}$	aerobic exercise	3 t/w
				CG: CT		0/19	35 ± 8			
Pressler* et al*. (2018) [33]	Germany	8 weeks	TAVI	EG: ET	17	5/5	82 ± 7	6MWT	endurance training, resistance training	None
				CG: CT		4/3	82 ± 7			
Zou* et al*. (2020) [48]	China	7 days	MVRS	EG: ET	80	29/11	53.21 ± 2.68	LVEF	abdominal breathing exercise, Passive limb functional movement	24 t/w
				CG: CT		27/13	53.78 ± 2.91			
Lin (2022) [41]	China	12 weeks	RVHD	EG: ET	84	23/19	70.68 ± 5.87	LVEF, SF-36	passive movement, walking	2–5 t/w
				CG: CT		25/17	71.05 ± 5.98			
Li (2021) [46]	China	7 days	AVHD	EG: ET	66	19/14	36.1 ± 6.9	LVEF, 6MWT	1–2 level: MOTO Med 3–4 level: aerobic exercise	7 t/w
				CG: CT		18/15	35.2 ± 6.8			
Li* et al*. (2021) [44]	China	12 weeks	VHD-HF	EG: ET	100	24/26	58.74 ± 2.84	LVEF, 6MWT	walking, resistance training	2–3 t/w
				CG: CT		23/27	55.62 ± 2.63			
Li* et al*. (2021) [45]	China	12 weeks	VHD-HF	EG: ET	80	19/21	59.8 ± 2.5	LVEF	walking, resistance training, cycle ergometer training	2–3 t/w
				CG: CT		22/28	59.8 ± 2.6			
Su* et al*. (2019) [39]	China	7 days	AVHD	EG: ET	108	32/22	55.6 ± 11.8	LVEF, 6MWT, SF-36	1–2 level: MOTO Med 3–4 level: aerobic exercise+ resistance training	7 t/w
				CG: CT		44/10	55.1 ± 11.7			
Cargnin* et al*. (2019) [34]	Brazil	4 weeks	HVRS	EG: ET	25	9/4	62.8 ± 8.8	6MWT, SF-36,	inspiratory muscle training	14 t/w
				CG: CT		6/6	60.1 ± 12.5			
Pressler* et al*. (2016) [32]	Germany	8 weeks	TAVI	EG: ET	27	7/6	81 ± 7	LVEF, 6MWT	cycle ergometer training, resistance training	3 t/w
				CG: CT		7/6	81 ± 5			
Rogers* et al*. (2018) [13]	UK	6 weeks	TAVI	EG: ET	27	6/7	81.21 ± 3.6	6MWT	aerobic exercise, functional exercise such as ‘sit to stand’, resistance training (both upper and lower body)	None
				CG: CT		6/8	82.92 ± 6.0			
Nilsson* et al*. (2019) [18]	Sweden	12 weeks	TAVR	EG: ET	12	5/1	58.5 ± 26.7	${\mathrm{VO}}_{\text{2peak}}$	cycle ergometer training	3 t/w
				CG: CT		4/2	65.5 ± 8.1			
Zhang (2022) [38]	China	2 weeks	AVHD	EG: ET	64	21/11	69.51 ± 13.42	LVEF, 6MWT	aerobic exercise, resistance training	7 t/w
				CG: CT		20/12	70.01 ± 12.91			
Liu* et al*. (2017) [40]	China	5–7 days	VHD	EG: ET	59	14/12	52.15 ± 11.37	SF-36	respiration training, active and passive movement on or near the bed, walking	14–21 t/w
				CG: CT		12/12	52.33 ± 10.42			
Liu* et al*. (2016) [49]	China	5–7 days	VHD	EG: ET	42	12/10	52.05 ± 13.22	LVEF, 6MWT	respiration training, active and passive movement on or near the bed, walking	14–21 t/w
				CG: CT		11/9	54.15 ± 10.06			
Li (2020) [42]	China	Non-disclosure	AVHD	EG: ET	60	16/14	68.12 ± 1.42	LVEF, 6MWT	walking, resistance training	None
				CG: CT		17/13	68.12 ± 1.41			

N, number; M, male; F, female; t/w, times/week; MVRS, mitral valve replacement surgery; EG, experimental group; ET, exercise intervention; CG, control group; CT, conventional intervention; TAVR, transcatheter aortic valve replacement; MVP, mitral valve prolapse; TAVI, transcatheter aortic valve implantation; RVHD, rheumatic valvular heart disease; AVHD, advanced valvular heart disease; VHD-HF, valvular heart disease with heart failure; HVRS, heart valve replacement surgery; VHD, valvular heart disease; LVEF, left ventricular ejection fraction; 6MWT, the six-minute walking test; SF-36, short-form 36-item; MOTO Med, an upper/lower limb rehabilitation equipment for people with movement restrictions and complements physical, ergo and sports therapy measures.

### 2.4 Quality Assessment

Two independent reviewers (LQZ and PP) assessed each included study’s risk of bias and quality using version 2 of the Cochrane risk-of-bias tool for randomised trials (RoB 2). This process included the following five domains: randomisation process, deviations from intended interventions, missing outcome data, outcome measurement, and reported result selection. The risk of bias in each domain was categorised as ‘low’, ‘some concerns’, or ‘high’, and the five domains determined the overall bias of each study. A superior expert (PZZ) resolved any disagreements.

### 2.5 Statistical Analysis

The meta-analysis was performed and the forest plots were created using Review Manager (RevMan, Version 5.4, The Cochrane Collaboration, Copenhagen, Denmark) software. Results with a *p*-value of <0.05 were considered statistically significant. Mean differences (MDs) with 95% confidence intervals (CIs) were calculated for each outcome indicator to compare the included studies. Statistical heterogeneity was assessed using the *$I^{2}$* statistic and Cochrane’ Q. *$I^{2}$* values >50% were considered high heterogeneity. The intervention’s pooled effect size was determined using a fixed effects model when *$I^{2}$* was <50% and a random effects model when *$I^{2}$* was ≥50%. Studies were excluded one by one to determine their effect on the results’ heterogeneity and the overall effect. Subgroup analyses were performed on intervention time, sample size, exercise combination mode, exercise frequency, and patient type. STATA (Version 17, StataCorp LLC, College Station Texas, College Station, TX, USA) software was used to produce funnel plots and Egger’s test. The symmetry of funnel plots was assessed through visual inspection and formally with Egger’s test to detect possible publication bias.

## 3. Results

### 3.1 Study Selection

**Fig. 1.**
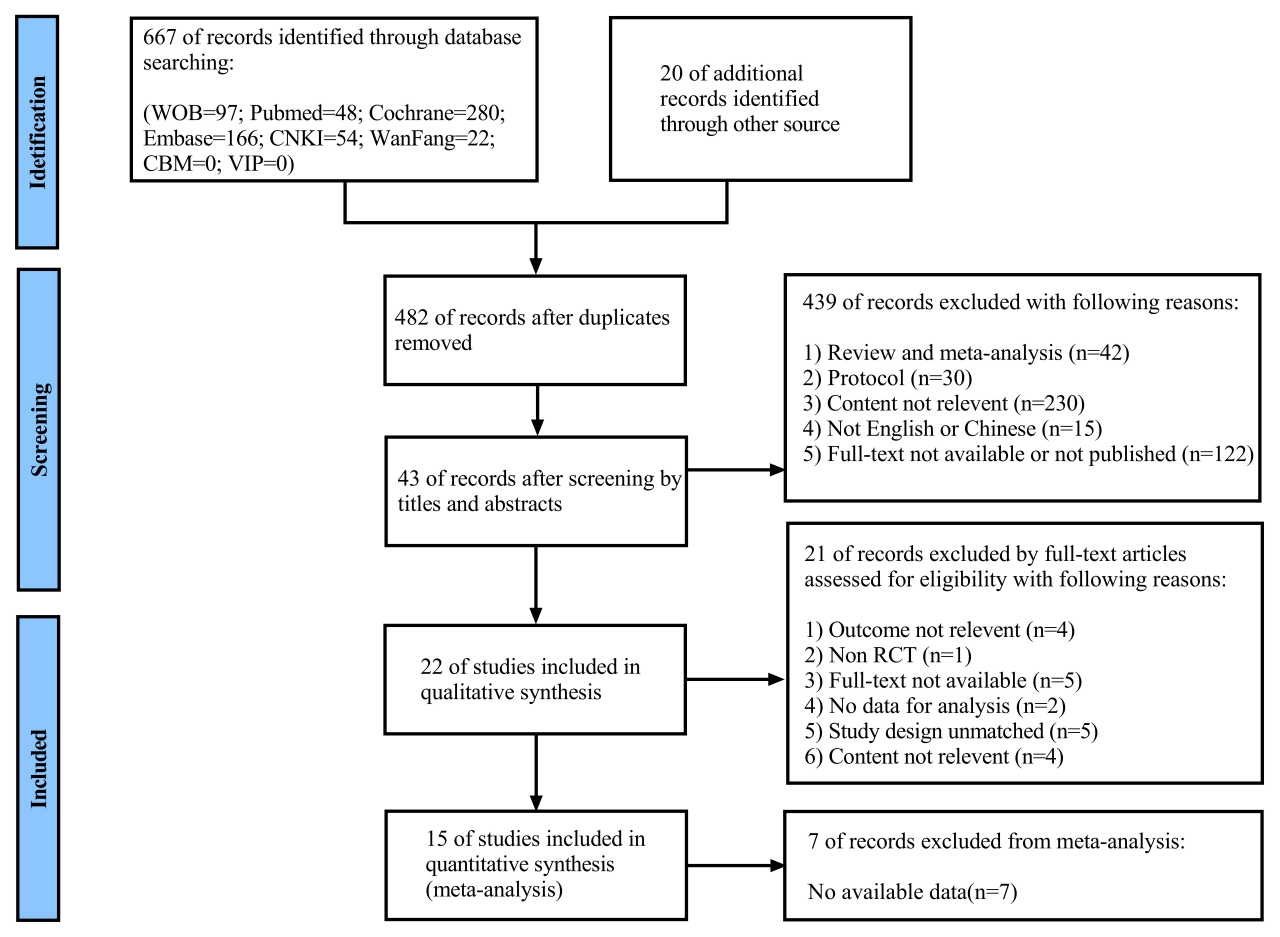
**Study selection flow chart**. WOB, web of science; CNKI, China National Knowledge Infrastructure; CBM, SinoMed; VIP, China Science and Technology Journal Database; RCT, randomized controlled trial.

The systematic search initially retrieved 687 articles from all sources: PubMed = 48, Cochrane = 280, Embase = 166, Web of Science = 97, CNKI = 54, WanFang = 22, and manual search = 20. Twenty-two studies [13, 18, 27, 32, 33, 34, 35, 36, 37, 38, 39, 40, 41, 42, 43, 44, 45, 46, 47, 48, 49, 50] were included in the final systematic review, of which 15 were included in the meta-analysis comparing exercise with no exercise in patients with VHD. The study selection process is shown in detail in Fig. 1.

### 3.2 Characteristics of the Studies

This study included 22 RCTs [13, 18, 27, 32, 33, 34, 35, 36, 37, 38, 39, 40, 41, 42, 43, 44, 45, 46, 47, 48, 49, 50] with 1520 subjects (Table 1), of which 731 were in the experimental group and 789 were in the control group. 869 (57.2%) were male, and 651 (42.8%) were female. In addition, no statistically significant differences were found between groups at baseline in each included study [13, 18, 27, 32, 33, 34, 35, 36, 37, 38, 39, 40, 41, 42, 43, 44, 45, 46, 47, 48, 49, 50]. These studies were mainly from China, two from the USA, two from Germany, one from the United Arab Emirates, one from Denmark, one from Brazil, one from the UK, and one from Sweden. However, it should be noted that mitral valve replacement (MVR) was performed in six of these studies, three with transcatheter aortic valve replacement (TAVR), three with transcatheter aortic valve implantation (TAVI), one with rheumatic VHD, and four with advanced VHD (AVHD).

The exercise frequency and duration varied among the RCTs, ranging from 2 to 30 times/week and 7d to 12w, respectively. In addition, the preoperative exercise rehabilitation usually lasted seven days, and the postoperative exercise intervention lasted mainly 12 weeks. The exercise form varied across the 22 included RCTs, including inspiratory muscle, aerobic, rehabilitation instrument-assisted, resistance, endurance, respiratory, passive, and functional training. Among them, aerobic exercise was mainly performed as walking, cycle ergometer training, and jogging.

This study’s outcome indicators were 6MWD, LVEF, and SF-36 (physical function [PF], social function [SF], bodily pain [BP] and mental health [MH] were selected). Of the 22 included articles, 18 reported 6MWD, 16 reported LVEF, and eight reported SF-36.

### 3.3 Quality Assessment

**Fig. 2.**
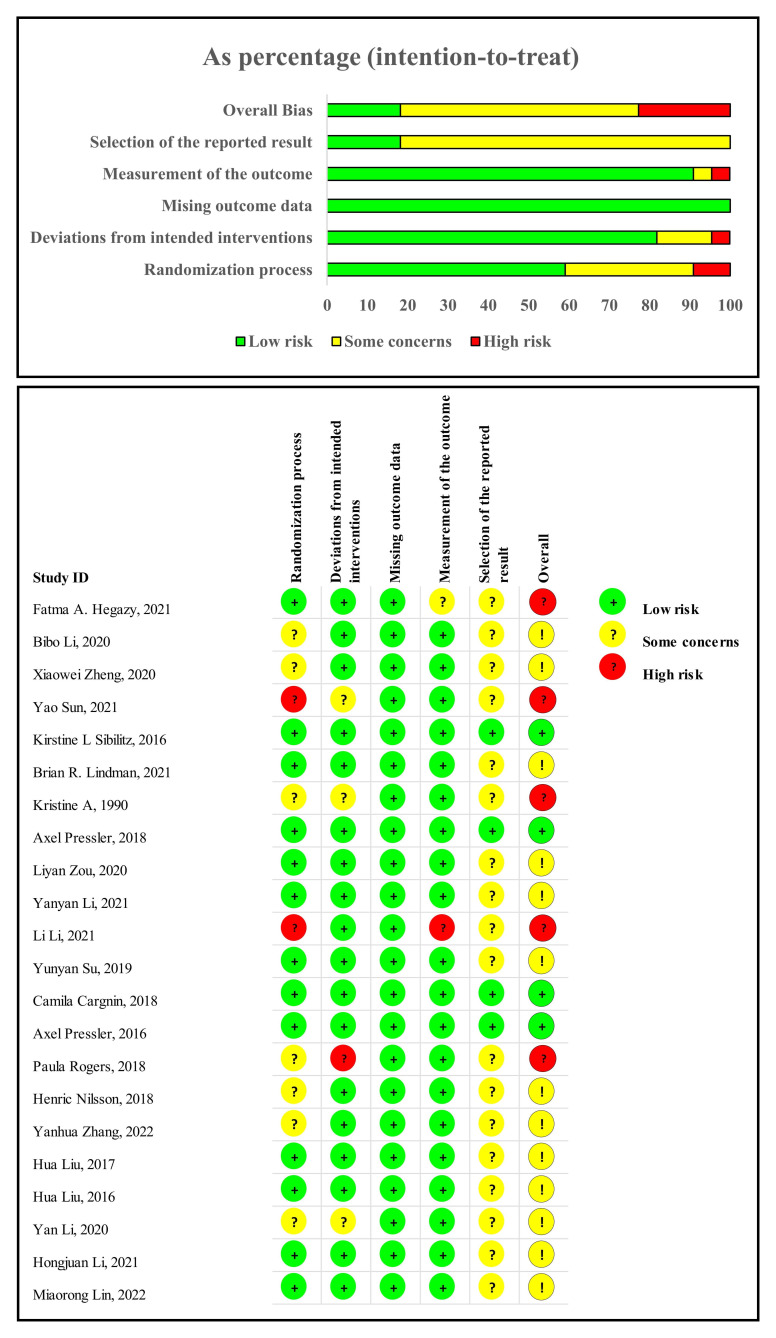
**Quality assessment**.

Two authors (LQZ and PP) independently reviewed and scored each study’s risk of bias and quality using RoB 2. The assessment results are presented in Fig. 2. Five studies [13, 36, 37, 43, 44] showed a high risk of bias mainly because of their randomisation process, deviations from intended interventions, and outcome measurement. Four studies [27, 32, 33, 34] showed a low risk of bias, and thirteen studies [18, 35, 38, 39, 40, 41, 42, 45, 46, 47, 48, 49, 50] showed a moderate risk of bias.

### 3.4 Sensitivity Analysis

Initially, the total heterogeneity for 6MWD, physical function (PF), social function (SF), bodily pain (BP) and LVEF was 38%, 46%, 59%, 69% and 81%. We conducted sensitivity analysis by eliminating articles one by one and found that the studies of Cargnin *et al*. [34], Liu *et al*. [49], Li *et al*. [50], and Zou *et al*. [48] had a significant influence on meta-analysis results. After removing these articles, the total heterogeneity for 6MWD, PF, SF, BP and LVEF dropped to 0%, 12%, 45%, 22%, and 66%, respectively. We speculate that this may be due to the exercise intervention method, the study by Cargnin *et al*. [34] only focused on respiratory muscle exercise without aerobic exercise, while others were the combination of a variety of exercise methods. For SF and BP, the study by Li *et al*. [50] not only conducted the exercise intervention but also conducted the motivational interview. The heterogeneity may even be related to the time of exercise intervention. The study of Liu *et al*. [49] intervened after surgery, while almost all studies on PF intervened before surgery.

### 3.5 Outcomes

#### 3.5.1 6MWD

**Fig. 3.**
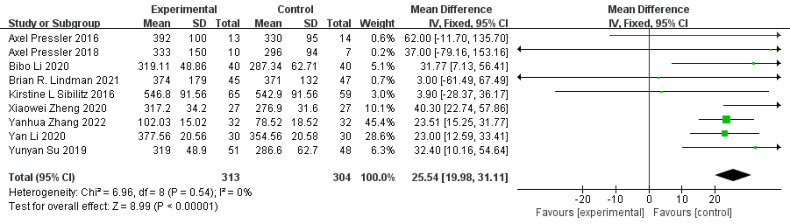
**Forest plot for 6MWD**. SD, standard deviation; IV, inverse variance; CI, confidence interval; 6MWD, six-minute walk test distance.

The 6MWT was the most common method to evaluate functional exercise capacity, used in nine studies [27, 32, 33, 35, 38, 39, 42, 47, 50]. We extracted data for the 6MWD from these nine studies and performed a meta-analysis. The fixed-effect model showed that exercise intervention significantly improved the functional capacity of patients with VHD compared to the control group (MD = 25.54, 95% CI: 19.98–31.11, *$I^{2}$* = 0%, *p* < 0.00001). The detailed results are shown in Fig. 3.

#### 3.5.2 LVEF

**Fig. 4.**
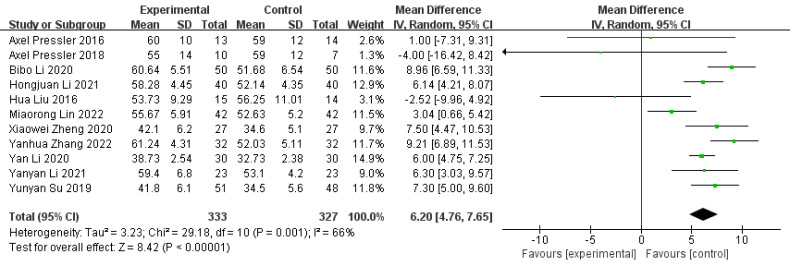
**Forest plot for LVEF. **SD, standard deviation; IV, inverse variance; CI, confidence interval; LVEF, left ventricular ejection fraction.

The normal range for LVEF is 50%~70%. It is an effective indicator of left ventricular systolic function, and its level reflects the state of cardiac function [51, 52, 53]. Eleven studies reported LVEF [32, 33, 38, 39, 41, 42, 45, 46, 47, 49, 50]. We used the random-effect model to pool the effect of the intervention on LVEF. The meta-analysis showed that exercise significantly improved LVEF in patients with VHD (MD = 6.20, 95% CI: 4.76–7.65, *$I^{2}$* = 66%, *p* < 0.00001). To explore the source of heterogeneity, we performed a subgroup analysis according to patient type, finding that exercise significantly improved LVEF in patients with MVRS (two studies [47, 50]; MD = 8.41, 95% CI: 6.54–10.27, *$I^{2}$* = 0%, *p* < 0.00001), VHD (two studies [41, 49]; MD = 2.52, 95% CI: 0.26–4.79, *$I^{2}$* = 49%, *p* = 0.03), AVHD (four studies [38, 39, 42, 46]; MD = 6.78, 95% CI: 5.83–7.73, *$I^{2}$* = 50%, *p* < 0.00001), and VHD with heart failure (one study [45]; MD = 6.14, 95% CI: 4.21–8.07, *p* < 0.00001). The detailed results are shown in Fig. 4.

#### 3.5.3 Quality of Life

**Fig. 5.**
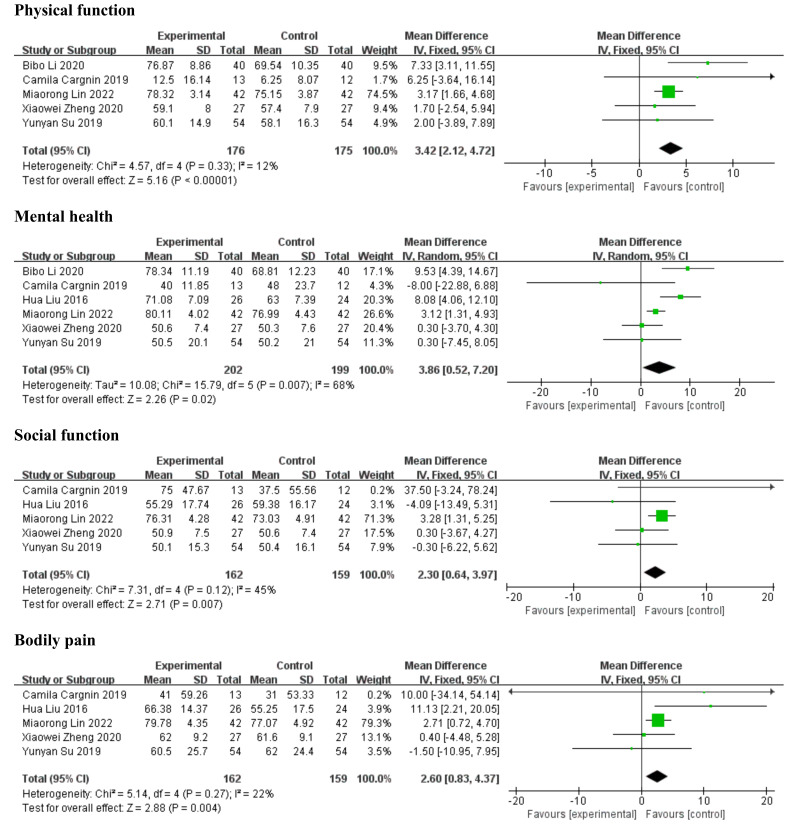
**Forest plots for quality of life. **SD, standard deviation; IV, inverse variance; CI, confidence interval.

When the term quality of life was introduced into the medical research field, it mainly referred to assessing the state of an individual’s physical, psychological, and social functions (i.e., quality of health) [54, 55, 56]. Therefore, we selected four dimensions of the SF-36 scale to reflect the effect of exercise on patients with VHD: MH, PF, SF, and BP. The detailed results are shown in Fig. 5.

3.5.3.1 PF

Five articles [34, 39, 41, 47, 50] reported PF and were included in the meta-analysis of exercise on the PF of patients with VHD. The fixed-effect model showed a significant improvement in PF (MD = 3.42, 95% CI: 2.12–4.72, *$I^{2}$* = 12%, *p* < 0.00001).

3.5.3.2 MH

Six studies [34, 39, 41, 47, 49, 50] reported MH and were included in the meta-analysis comparing MH between the exercise and control groups. The random-effect model showed that exercise significantly improved MH in patients with VHD compared to the control group (MD = 3.86, 95% CI: 0.52–7.20, *$I^{2}$* = 68%, *p* = 0.020). To explore the source of heterogeneity, we performed subgroup analyses according to patient type, time of intervention, and exercise frequency; however, no sources of heterogeneity were found.

3.5.3.3 SF

Five studies [34, 39, 41, 47, 49] reported SF and were included in the meta-analysis. We used the fixed-effect model to pool the effect of the intervention on SF. The meta-analysis showed that exercise significantly improved the SF of patients with VHD compared to the control group (MD = 2.30, 95% CI: 0.64–3.97, *$I^{2}$* = 45%, *p* = 0.007).

3.5.3.4 BP

Five studies [34, 39, 41, 47, 49] reported BP and were included in the meta-analysis. We used the fixed-effect model to pool the effect of the intervention on BP. The meta-analysis showed that exercise significantly improved BP in patients with VHD compared to the control group (MD = 2.60, 95% CI: 0.83–4.37, *$I^{2}$* = 22%, *p* = 0.004).

### 3.6 Publication Bias

**Fig. 6.**
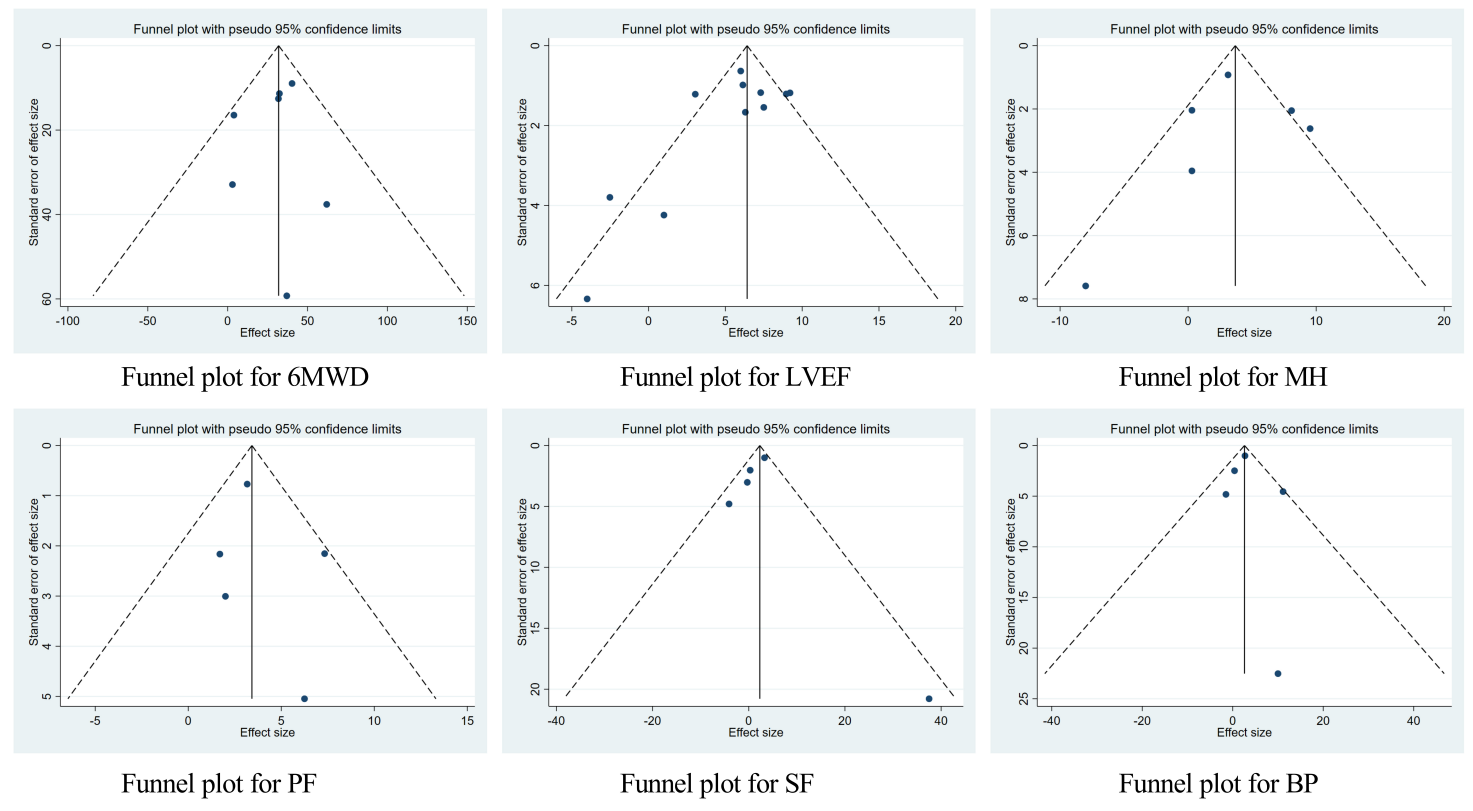
**Funnel plots of synthesis outcomes. **6MWD, the six-minute walk test distance; LVEF, left ventricular ejection fraction; MH, mental health; PF, physical function; SF, social function; BP, bodily pain.

The funnel plots of synthesis outcomes showed a symmetric distribution (Fig. 6). The results of Egger’s test showed no publication biases (all *p* > 0.05).

## 4. Discussion

To our knowledge, this is the first systematic review investigating exercise effects on cardiopulmonary function in patients with VHD. This study had three main findings. First, compared to conventional therapy, exercise was significantly associated with improved 6MWD in patients with VHD. Second, the exercise intervention significantly increased LVEF, reflecting a significant improvement in cardiac function. Third, exercise positively affected quality of life by improving PF, SF, and MH and reducing BP. The heterogeneity of our results was low. It should be noted that Egger’s test showed no publication bias in the improvement effect of the exercise intervention on 6MWD, LVEF and SF-36, and the meta-analysis results were stable.

In particular, there is little evidence for applying perioperative exercise in patients with VHD, and only a few small exploratory studies have been reported [13, 18, 27, 32, 33, 34, 35, 37]. Some physicians have guided exercise rehabilitation in patients with VHD based on their clinical experience or exercise prescription in other CVD populations, such as patients with ischaemic heart disease. This study included 22 RCTs with 1520 participants, which may provide new evidence for these patients.

Cardiopulmonary exercise testing (CPET) provides an accurate assessment of physiological responses induced by exercise and is extremely important in the clinical environment, especially for evaluating peak oxygen uptake (${\mathrm{VO}}_{\text{2peak}}$) [57]. Despite its confirmed validity, applying CPET remains complex, expensive, and dependent on highly trained professionals [58]. Furthermore, CPET may be poorly tolerated by elderly patients or those with comorbidities [59]. Moreover, CPET does not represent functional capacity in real life [59]. Instead, the 6MWT is a simple test that requires no specialised equipment or advanced training for physicians and assesses an individual’s submaximal level of functional capacity while walking on a flat, hard surface for 6 minutes [60]. The 6MWD is often used as an index of CRF and has been validated in several populations of patients with chronic diseases [61]. Increases in fitness are independently associated with reduced mortality and morbidity and improved quality of life [62]. In addition, a study found that a reduced 6MWD in patients with heart failure was associated with poor quality of life and a worse prognosis [63]. Therefore, our results show that patients with VHD had increased 6MWD after exercise, potentially benefiting prognosis. In addition, the results of a previous meta-analysis by Ribeiro *et al*. [29] showed CR improved exercise tolerance and functional independence measured by 6MWT, consistent with our findings.

Improved LVEF is very important and necessary for patients with VHD. LVEF is the most commonly used indicator during echocardiography assessment, which shows signs of left ventricular dysfunction in patients with VHD [64]. A low LVEF was associated with an increased risk of poor perioperative prognosis. Baron *et al*. [65] showed that reduced LVEF was common in patients after TAVI and that LVEF values were strongly associated with mortality and recurrent heart failure in patients with VHD at one year postoperatively. In addition, Borer *et al*. [66] concluded that LVEF recovery generally takes three years after aortic valve replacement in patients with aortic insufficiency. Our meta-analysis showed that LVEF was significantly improved in patients with VHD after the exercise intervention, which would have an integral role in improving their clinical prognosis after surgery. In addition, the improved prognosis of patients with VHD after surgery due to increased LVEF may be associated with enhanced body metabolism from increased stroke volume and oxygen content [67].

However, we observed heterogeneity among the included articles for LVEF. In the examination of heterogeneity sources for improvements in LVEF, no significant influences were found for intervention time, sample size, exercise combination mode, and exercise frequency. However, when heterogeneity was examined from the perspective of patient type, it was found to be the apparent heterogeneity source. Based on subgroup analysis of patient types, we found that the improvements in LVEF of patients with TAVI due to the exercise intervention were nonsignificant, which may be explained by patients with TAVI not requiring an open chest procedure. Consequently, their surgical wounds are smaller and their baseline values are better than those of patients undergoing other forms of open chest surgery. Therefore, there is little change. However, in patients with more severe valve damage, such as AVHD, or after open-chest surgery, such as MVRS, the results showed that exercise had a greater effect on their LVEF.

As an evaluation scale for quality of life, the SF-36 subjectively measures an individual’s perception of various life aspects. It is a comprehensive indicator of an individual’s health status, reflecting the physical, psychological, and social health of patients with VHD after surgery. In our study, the SF-36 was selected to evaluate the effect of exercise interventions on the quality of life of patients with VHD. Our results indicated that patients with VHD showed significant improvements in PF, SF, and BP. MH may be improved after exercise since there was high heterogeneity in the included RCTs. Oterhals *et al*. [68] found that patients’ physical and mental health after aortic valve replacement was worse than the general population. An RCT by Rêgo *et al*. [69] showed a good correlation between physical and mental health and exercise capacity, consistent with the results of our meta-analysis, which showed significant improvements in outcomes, such as 6MWD and PF, in patients with VHD.

For heterogeneity in quality of life, we also performed a subgroup analysis of MH by patient type; however, it was not a source of heterogeneity. We hypothesise that this may be because our results are derived from a subjective perception scale. Each patient has a different feeling about the same thing, and self-reported outcomes are by their nature subjective and, therefore, at risk of recall bias.

There are multiple benefits from exercise for patients with VHD. A 12-week aerobic exercise programme for symptomatic women with mitral valve prolapse showed that the exercise group had improved functional capacity and well-being compared to the control group, with an associated reduction in anxiety scores and the frequency of symptoms [36]. Exercise-based rehabilitation before cardiac surgery was associated with a lower risk of postsurgical complications and reduced hospital stay length [70]. Similarly, when initiated early after cardiac surgery, aerobic exercise improved functional capacity and quality of life [71]. Consistent with our findings, exercise improved cardiopulmonary function, including exercise tolerance and myocardial contractility, in patients with VHD.

There is a dearth of studies investigating the natural history of VHD in exercising individuals, and exercise recommendations are primarily based on expert consensus [31]. Given the well-established benefits of physical activity on cardiovascular health and overall well-being, patients with VHD of all severities should be encouraged to participate in regular physical activity and exercise [31]. Chatrath *et al*. [11] recommended that all patients with VHD should be encouraged to avoid sedentary behaviour by engaging in at least 150 minutes of physical activity every week, including strength training. In our study, most participants were perioperative patients and therefore did not meet the above recommendations for exercise duration, although these recommendations can be implemented in these patients later in their recovery.

Our study also found that most exercise programs used in the included RCTs were combination types, not restricted to a single form, with aerobic exercise combined with resistance training notably more common. Many studies added respiratory training and passive exercise for perioperative patients. Incorporating strategies such as early mobilisation and respiratory-based exercises may provide some benefits in preventing postoperative pulmonary complications [71]. Our meta-analysis showed that exercise improved cardiopulmonary function and quality of life in patients with VHD, which may be related to the fact that moderate exercise may reduce CVD risk by increasing myocardial blood flow reserve, improving vascular endothelial cell function, improving skeletal muscle oxygen uptake and utilisation, and reducing mitochondrial damage in cardiomyocytes, thereby promoting body metabolism [67, 72]. In addition, a study indicated that exercise after TAVI improved functional capacity, even exceeding the effects of the TAVI surgery [73], consistent with our results.

Since most existing research is based on clinical patients, exercise and its effects after discharge were not considered. In future studies, it is necessary to evaluate the long-term effects of exercise and its effects on patients who do not undergo surgery. Secondly, one study found that patients with chronic heart failure had a shorter step length and walked more slowly during the 6MWT than controls [74]. Therefore, when using the 6MWT to assess cardiopulmonary function, future studies should consider step length, age, height, and even body mass index because they are predictors of 6MWD [75]. Thirdly, exercise-based CR is a beneficial tool for the secondary prevention of CVD, albeit with low participation rates [76]. Telerehabilitation, intergrading mobile technologies and wireless sensors, may advance cardiac patients’ adherence. Future studies could consider telerehabilitation as an alternative to contemporary centre/community-based CR, integrating real-time supervision and group-based exercise sessions in cardiac telerehabilitation.

### Strengths and Limitations

The strength of this study is its originality since it is the first systematic review with meta-analysis investigating improvements in cardiopulmonary function due to exercise and even the impact of exercise on quality of life. In addition, this study included all relevant literature published from inception to January 2023. Therefore, this study’s larger sample than similar previous studies indicates that it is more comprehensive and representative. Furthermore, this study’s heterogeneity is low, while that of similar previous studies was high. Finally, our results may be more generalisable since we included a wide range of ages and both men and women.

This systematic review also had some limitations. Firstly, we did not exclude patients with CVD and VHD, who may have more complex changes in functional capacity and quality of life than patients with VHD alone. However, we performed a subgroup analysis based on the types of patients with VHD to compare the difference. Secondly, most included studies were limited to patients’ perioperative rehabilitation exercises. Therefore, patients with mild VHD were not considered. Future studies are expected to further explore the appropriate exercise prescription for patients with different VHD types, considering the severity of their disease simultaneously. Thirdly, some of our meta-analysis results showed high heterogeneity, although no source of heterogeneity was found through subgroup analysis, which needs further discussion in future research.

## 5. Conclusions

In summary, exercises involving walking, resistance, and even respiratory training for patients with VHD significantly improve cardiopulmonary function, PF, and SF; reduce BP; and potentially improve MH. This study supports the literature regarding the benefits of exercise on cardiopulmonary function, providing an evidence-based basis for better recovery in patients with VHD. Prospective studies should further investigate the most appropriate exercise plan for patients with VHD, especially a long-term program.

## Abbreviations

VHD, valvular heart disease; RHD, rheumatic heart disease; AS, aortic stenosi; AVD, aortic valve disease; CR, cardiac rehabilitation; CHD, coronary heart disease; HF, heart failure; PROSPERO, prospective register of systematic review; PRISMA, preferred reporting items for systematic reviews and meta-analysis; WOB, web of science; CNKI, China National Knowledge Infrastructure; VIP, China Science and Technology Journal Database; CBM, SinoMed; RCT, randomized controlled trial; 6MWD, 6-minute walk test distance; 6MWT, 6-minute walk test; LVEF, left ventricular ejection fraction; SF-36, short form 36-item health survey; Cochrane CENTRAL, cochrane central register of controlled trials; ROB, risk-of-bias; MDs, mean differences; CIs, confidence intervals; EG, experimental group; CG, control group; USA, the United States of America; UAE, United Arab Emirates; UK, the United Kingdom; MVR, mitral valve replacement; TAVR, transcatheter aortic valve replacement; TAVI, transcatheter aortic valve implantation; AVHD, advanced valvular heart disease; PF, physical function; SF, social function; BP, bodily pain; MH, mental health; CPET, cardiopulmonary exercise testing; MVRS, mitral valve replacement surgery; MVP, mitral valve prolapse.

## Supplementary Material

Supplementary material associated with this article can be found, in the online version, at https://doi.org/10.31083/j.rcm2408237.
