# Correction: Zeng et al. Exercise as the Key to Improve Cardiopulmonary Function in Patients With Valvular Heart Disease: A Systematic Review and Meta-Analysis. Reviews in Cardiovascular Medicine. 2023; 24(8): 237

**DOI:** 10.31083/RCM58936

**Published:** 2026-10-16

**Authors:** Liqing Zeng, Peng Pi, Peizhen Zhang, Yu Zhu, Lumeng Yang, Chen Wang

**Affiliations:** ^1^School of Sports Medicine and Rehabilitation, Beijing Sport University, 100084 Beijing, China; ^2^Department of Rehabilitation Medicine, Linfen Central Hospital, 041000 Linfen, Shanxi, China

The authors would like to correct the directional labels at the bottom of the forest plots in Figs. 3,4,5 of the article entitled “Exercise as the Key to Improve Cardiopulmonary Function in Patients With Valvular Heart Disease: A Systematic Review and Meta-Analysis” [1]. In the original figures, the labels “Favours experimental” and “Favours control” were inadvertently reversed during figure generation using Review Manager 5 (RevMan 5), version 5.4 (The Cochrane Collaboration, Copenhagen, Denmark). In the corrected figures, “Favours control” is shown on the left side of the horizontal axis and “Favours experimental” on the right side.

In Fig. 3, the positions of “Favours (experimental)” and “Favours (control)” have been exchanged.

The original Fig. 3:

**Fig. 3.**
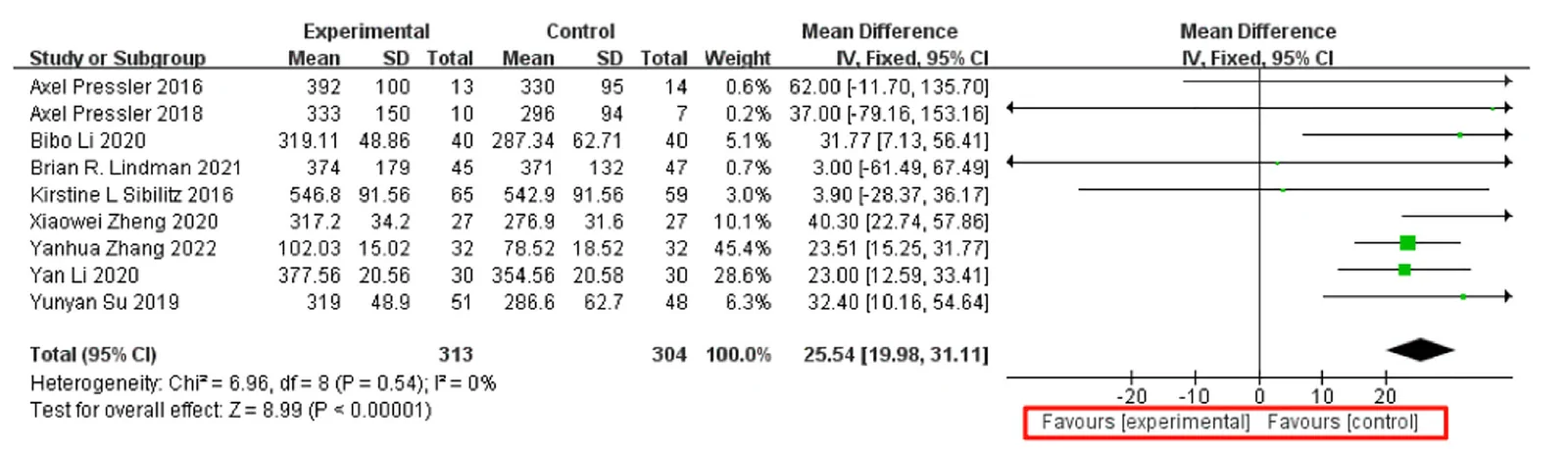
**Forest plot of the effect of exercise on 6MWD**. SD, standard deviation; IV, inverse variance; CI, confidence interval; 6MWD, six-minute walk test distance.

The corrected Fig. 3:

**Fig. 3.**
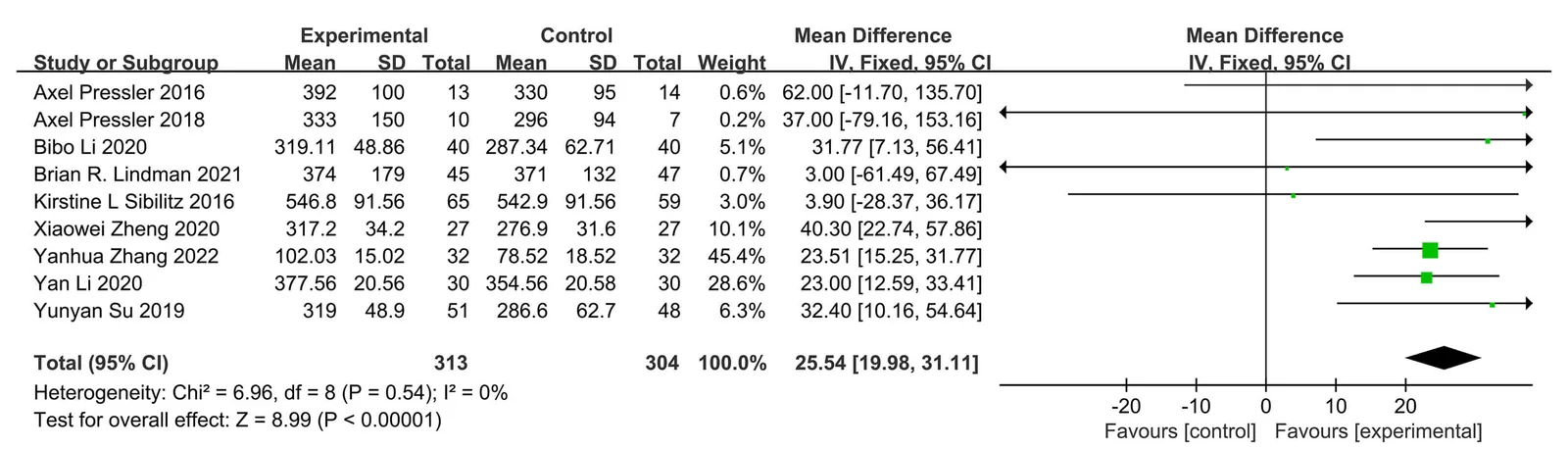
**Forest plot of the effect of exercise on 6MWD**. SD, standard deviation; IV, inverse variance; CI, confidence interval; 6MWD, six-minute walk test distance.

In Fig. 4, the positions of “Favours (experimental)” and “Favours (control)” have been exchanged.

The original Fig. 4:

**Fig. 4.**
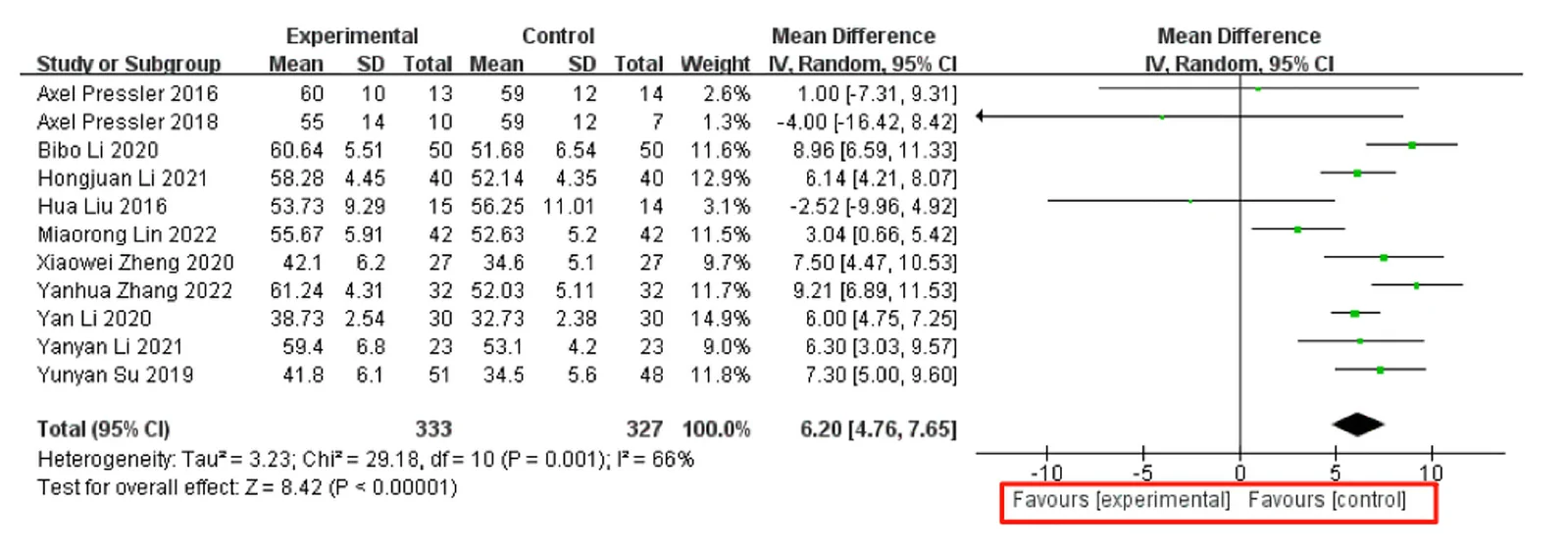
**Forest plot of the effect of exercise on LVEF**. LVEF, left ventricular ejection fraction.

The corrected Fig. 4:

**Fig. 4.**
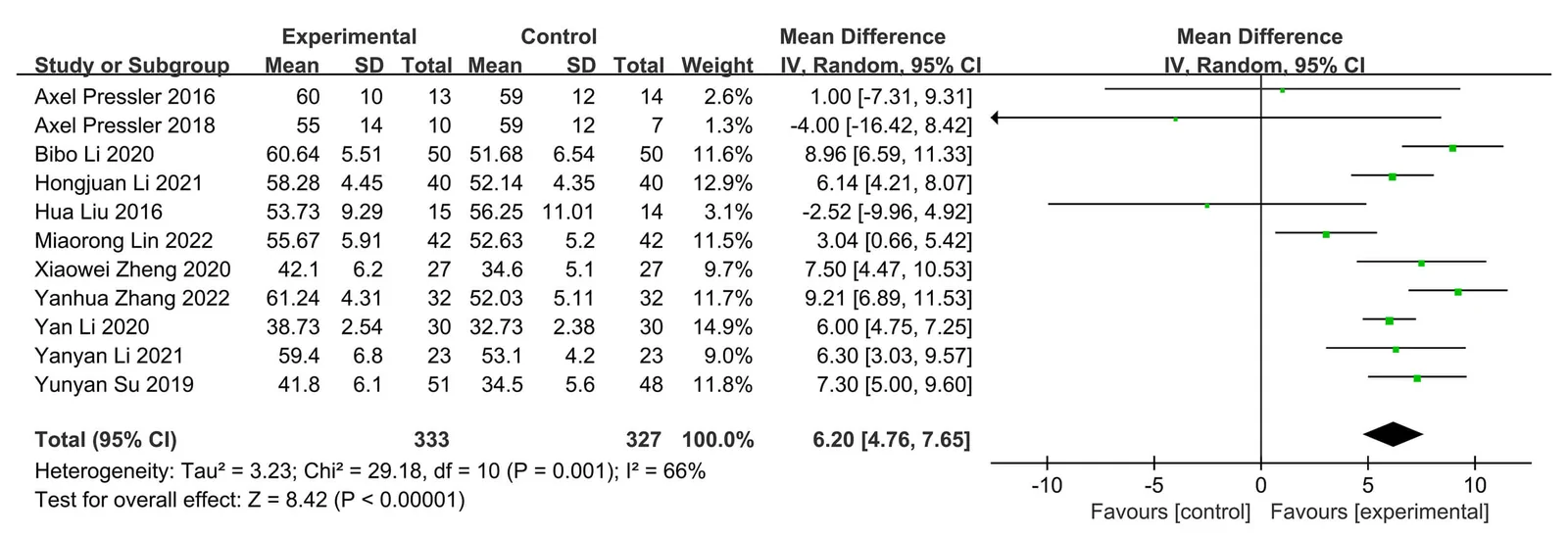
**Forest plot of the effect of exercise on LVEF**. SD, standard deviation; IV, inverse variance; CI, confidence interval; LVEF, left ventricular ejection fraction.

In Fig. 5, the positions of “Favours (experimental)” and “Favours (control)” have been exchanged.

The original Fig. 5:

**Fig. 5.**
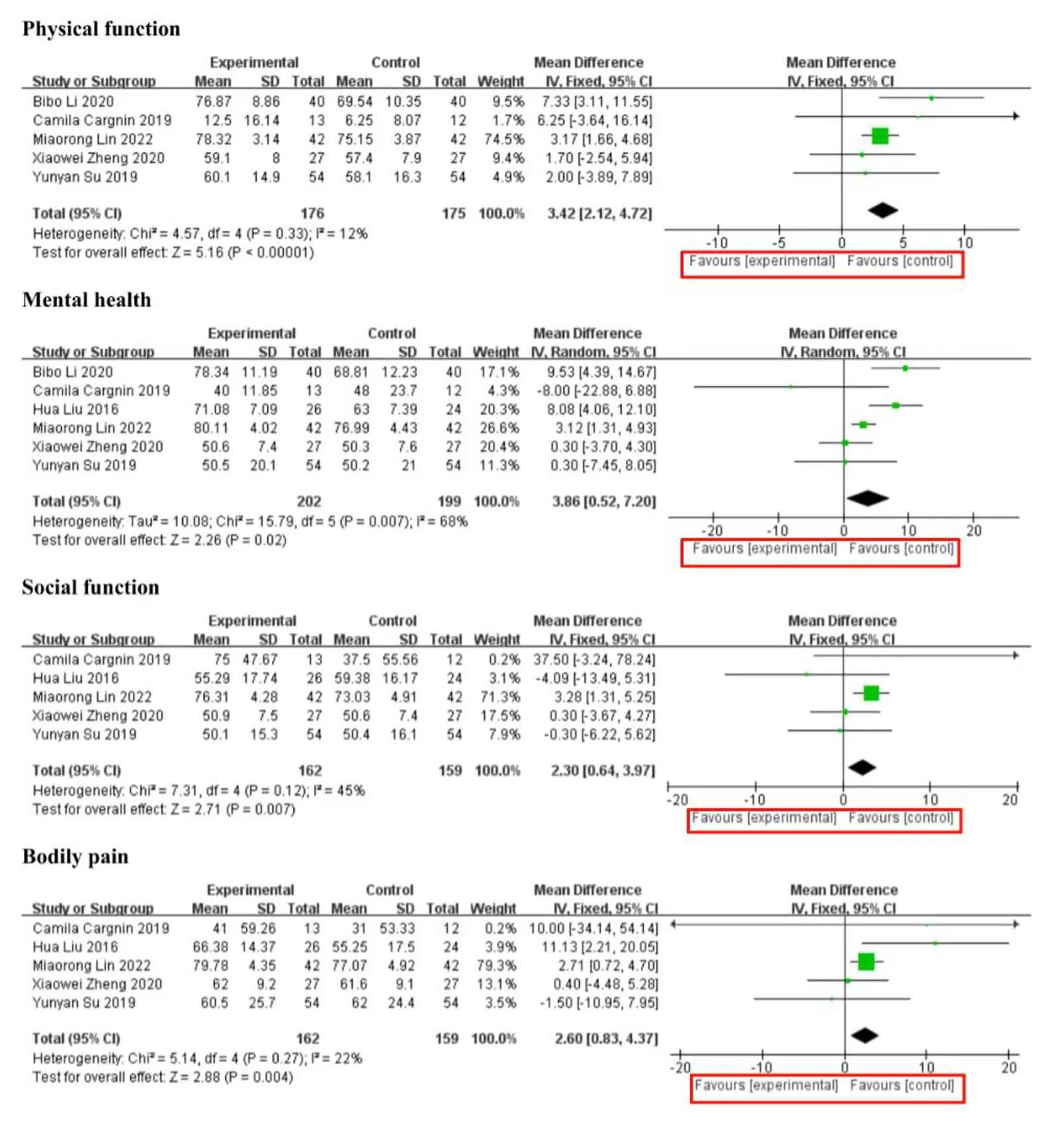
**Forest plots of the effect of exercise on quality of life**.

The corrected Fig. 5:

**Fig. 5.**
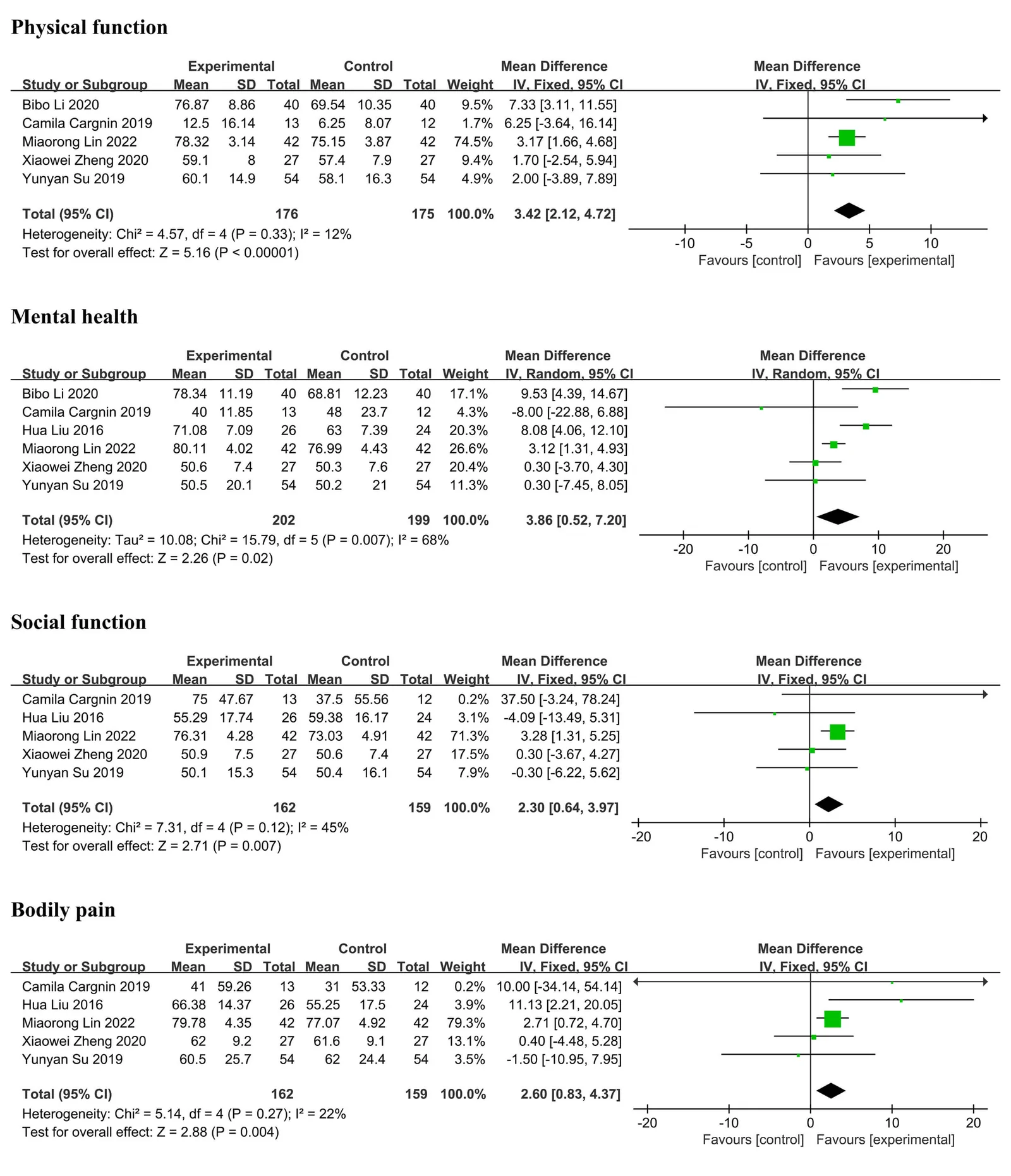
**Forest plots of the effect of exercise on quality of life**. SD, standard deviation; IV, inverse variance; CI, confidence interval.

The statistical data presented in the figures and text, including the mean differences, confidence intervals, and *p*-values, were correctly calculated and interpreted. Therefore, these corrections do not affect the results, discussion, or conclusions of the article.

This has been corrected as of 28 July 2026. The authors apologize for these errors. This correction has been approved by the Editor-in-Chief of the journal.
